# Increased Adiposity in Annexin A1-Deficient Mice

**DOI:** 10.1371/journal.pone.0082608

**Published:** 2013-12-02

**Authors:** Rand T. Akasheh, Maria Pini, Jingbo Pang, Giamila Fantuzzi

**Affiliations:** Department of Kinesiology and Nutrition, University of Illinois at Chicago, Chicago, Illinois, United States of America; Dasman Diabetes Institute, Kuwait

## Abstract

Production of Annexin A1 (ANXA1), a protein that mediates the anti-inflammatory action of glucocorticoids, is altered in obesity, but its role in modulation of adiposity has not yet been investigated. The objective of this study was to investigate modulation of ANXA1 in adipose tissue in murine models of obesity and to study the involvement of ANXA1 in diet-induced obesity in mice. Significant induction of ANXA1 mRNA was observed in adipose tissue of both C57BL6 and Balb/c mice with high fat diet (HFD)-induced obesity *versus* mice on chow diet. Upregulation of ANXA1 mRNA was independent of leptin or IL-6, as demonstrated by use of leptin-deficient *ob/ob* mice and IL-6 KO mice. Compared to WT mice, female Balb/c ANXA1 KO mice on HFD had increased adiposity, as indicated by significantly elevated body weight, fat mass, leptin levels, and adipocyte size. Whereas Balb/c WT mice upregulated expression of enzymes involved in the lipolytic pathway in response to HFD, this response was absent in ANXA1 KO mice. A significant increase in fasting glucose and insulin levels as well as development of insulin resistance was observed in ANXA1 KO mice on HFD compared to WT mice. Elevated plasma corticosterone levels and blunted downregulation of 11-beta hydroxysteroid dehydrogenase type 1 in adipose tissue was observed in ANXA1 KO mice compared to diet-matched WT mice. However, no differences between WT and KO mice on either chow or HFD were observed in expression of markers of adipose tissue inflammation.

These data indicate that ANXA1 is an important modulator of adiposity in mice, with female ANXA1 KO mice on Balb/c background being more susceptible to weight gain and diet-induced insulin resistance compared to WT mice, without significant changes in inflammation.

## Introduction

Obesity is a worldwide epidemic and a major risk factor for several morbidities, partly through induction of chronic inflammation [1]. Accumulation of fat in the visceral (VAT) rather than the subcutaneous (SAT) adipose tissue is associated with higher inflammation and increased risk of obesity-related diseases [2].

Annexin A1 (ANXA1) is the first identified member of the annexin family of proteins that regulate various cellular functions and bind to phospholipids in a calcium-dependent manner [3]–[6]. Glucocorticoids (GC) regulate production of ANXA1, which in turn mediates at least part of GC's anti-inflammatory actions [7]. Administration of ANXA1 or its agonists is an effective anti-inflammatory therapy [8], [9], whereas ANXA1 deficiency or antagonists lead to more severe inflammation in various experimental models and blunt the anti-inflammatory effects of GC [8], [10], [11].

Limited and partly controversial evidence indicates a potential role for ANXA1 in obesity and diabetes. Transcriptome analysis of human adipose tissue reveals increased ANXA1 expression in response to obesity [12]. In contrast, plasma ANXA1 levels are inversely correlated with markers of adiposity and inflammation in obese subjects [13]. In humans, the peroxisome proliferator-activated receptor-gamma (PPARγ) agonist rosiglitazone upregulates ANXA1 in adipose tissue, a response that is in line with the anti-inflammatory, insulin-sensitizing actions of this compound [14]. However, ANXA1 is also upregulated in leukocytes after a glucose load or exposure to TNFα, which induces insulin resistance [15]. Therefore, the role and regulation of ANXA1 in the context of obesity and diabetes remain to be elucidated.

In the present study we investigated the modulation of ANXA1 in adipose tissue in models of diet-induced (DIO) and genetic obesity, and also studied whether ANXA1 participates in modulation of adiposity, glucose metabolism and obesity-associated inflammation using a model of DIO. Due to the pro-lipolytic and anti-inflammatory effects of ANXA1 [16], we hypothesized that ANXA1 KO mice would develop increased adiposity accompanied by heightened inflammation in response to DIO. To test this hypothesis, we studied female Balb/c mice, that are resistant to DIO [17], to investigate whether ANXA1 deficiency alters their phenotype towards increased adiposity, inflammation and insulin resistance.

## Methods

### Ethics statement

Mouse studies were approved by the Animal Care and Use Committee at the University of Illinois at Chicago under protocol A10-008.

### Animals

Breeding pairs of ANXA1 KO mice in a Balb/c background were a kind gift of Dr. Asma Nusrat (Emory University, Atlanta, GA), generated as described [10]. Balb/c WT as well as C57BL6 WT, IL-6 KO and *ob/ob* mice were purchased from the Jackson Laboratory (Bar Harbor, ME). For induction of DIO, mice received HFD (60% of calories from fat, Research Diets, New Brunswick, NJ) for 5–14 weeks. Control groups received chow diet. Water and food were available *ad libitum*. Mice were weighed weekly.

### Evaluation of adiposity

Dual-energy X-ray Absorptiometry (DXA) was used to quantify body composition. The whole body was included in the analysis region, except for the head and tail.

### Adipocyte size

Adipocyte size was measured using ImageJ software. Two hundred adjacent cells were analyzed from a total of three different hematoxylin-eosin-stained sections from each mouse. Data are expressed as median adipocyte area in µm^2^.

### Glucose tolerance test (GTT)

Five-hour fasting blood glucose was measured at time 0, then mice received an ip injection of 1 gram of glucose per Kg of body weight. Blood glucose was measured from a tail nick at time 15, 30, 60, and 90 minutes.

### Insulin tolerance test (ITT)

Blood glucose of fed mice was measured at time 0, then mice received an ip injection of 1 unit of insulin per Kg of body weight. Blood glucose was measured from a tail nick at time 15, 30, 60, and 90 minutes.

### Flow cytometry

Evaluation of ANXA1 expression in the stromovascular fraction (SVF) obtained from VAT by collagenase digestion was evaluated by flow cytometry as previously described [18] using an antibody (AF-3770) from R&D Systems (Minneapolis, MN).

### Measurement of circulating mediators

Plasma levels of leptin, adiponectin, insulin and corticosterone were measured using ELISA kits from R&D Systems, Alpco (Salem, NH) and Cayman Chemicals (Ann Arbor, Mi), respectively.

### Gene expression analysis

To quantify gene expression, qPCR was performed using probes for the TaqMan system to measure acetyl-CoA carboxylase (ACC), adiponectin, ANXA1, chemokine (C-C motif) ligand 2 (CCL2), cluster of differentiation-68 (CD68), fatty acid synthase (FAS), formyl peptide receptor -2 (FPR2), glucose 6-phosphatase (G6Pase), galectin-12, interleukin-1β (IL-1β), IL-6, IL-10, leptin, PPAR-γ, phophoenolpyruvate carboxykinase (PEPCK), and sterol regulatory element-binding transcription factor 1 (SREBF1). Quantification of adipose triglyceride lipase (ATGL), 11-beta-hydroxysteroid dehydrogenase (11βHSD1), and hormone sensitive lipase (HSL) was performed using the SYBR Green system. Target gene expression values were normalized to glyceraldehyde 3-phosphate dehydrogenase (GAPDH) as a reference gene, and analyzed using the ΔΔ^CT^ method.

### Western blotting

Samples of VAT were homogenized, lysed in RIPA buffer, centrifuged and normalized by protein concentration. After gel electrophoresis and transfer, membranes were blocked in 1% BSA and incubated overnight at 4°C with anti-HSL and anti-pHSL antibodies (Cell Signaling Technology, Danvers, MA). Membranes were incubated at room temperature for 1 hour with an HRP-conjugated secondary antibody, washed and detected by enhanced chemiluminescence (Thermo Scientific, Pittsburgh PA).

### Statistical analysis

Statistical significance was defined as p value of less than 0.05. Analysis of Variance (ANOVA) and Student t-test were used to test for significance where appropriate using MedCalc software (Mariakerke, Belgium).

## Results

### Expression of ANXA1 in adipose tissue

**Figure 1:**
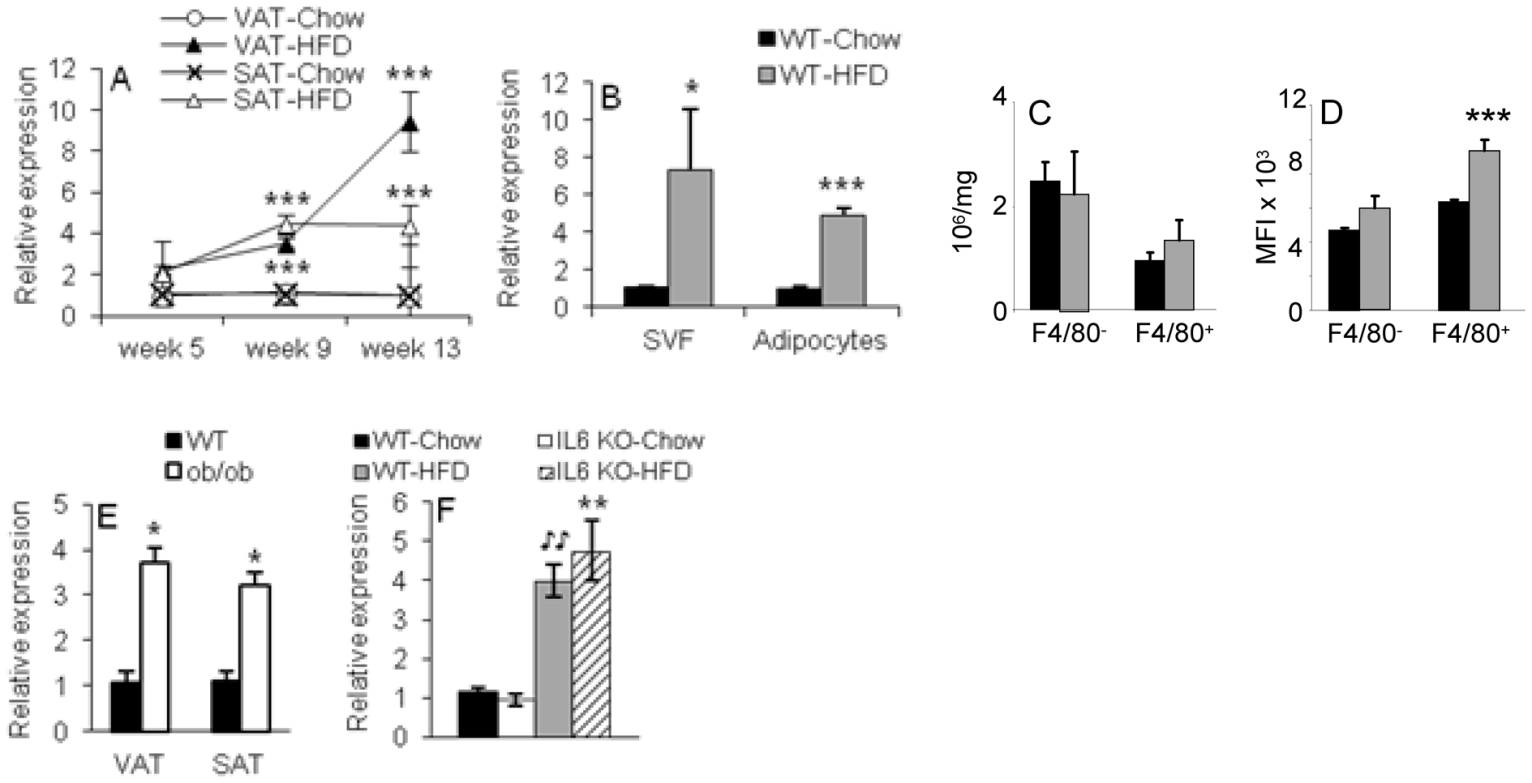
Modulation of ANXA1 expression in adipose tissue. Panel A: Time course of ANXA1 mRNA expression in VAT and SAT of male C57BL6 mice fed chow or HFD for 5, 9 or 13 weeks. Data are expressed as fold difference vs. the respective tissue in chow mice at 5 weeks. ***p<0.001 vs. respective tissue in chow mice. Panel B: Expression of ANXA1 mRNA in VAT SVF and adipocytes of male C57BL6 mice fed chow or HFD for 13 weeks (n = 9). *p<0.05, ***p<0.001 vs. respective fraction in chow mice. Panels C and D: Number (Panel C) and Mean Fluorescence Intensity (Panel D) of ANXA1^+^ cells per mg of tissue in the F4/80^-^ and F4/80^+^ populations from VAT of male C57BL6 mice fed chow or HFD for 13 weeks evaluated by flow cytometry (n = 5). ***p<0.001 vs. respective cell population in chow mice. Panel E: Expression of ANXA1 mRNA at eight weeks of age in VAT of male WT and *ob/ob* mice fed chow diet (n = 3). Panel F: Expression of ANXA1 mRNA in VAT of male WT and IL-6 KO mice fed chow or HFD for 13 weeks (n = 3 – 5). **p<.005 vs. WT-HFD, ^
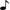
^p<.05 vs. WT-Chow. Data are mean ± SEM.

To investigate whether obesity induced by HFD alters expression of ANXA1 in adipose tissue, we used male C57BL6 mice, a strain that is highly susceptible to DIO [18]. Expression of ANXA1 mRNA was significantly elevated in VAT and SAT of male C57BL6 mice after 9 and 13 weeks of HFD compared to chow groups (Figure 1A). During this time course, male C57BL6 mice on HFD became progressively obese and developed VAT inflammation, as expected [18]. Feeding a HFD lead to increased ANXA1 expression in both the SVF and the adipocyte fraction in VAT of HFD compared to chow groups, indicating upregulation in both the adipocyte and non-adipocyte components of adipose tissue (Figure 1B). Further characterization of ANXA1^+^ cells in the VAT SVF indicated production by both F4/80^+^ (macrophages) and F4/80^-^ cells, with comparable numbers of ANXA1^+^ cells per mg of tissue in chow and HFD mice, but significantly elevated mean fluorescence intensity in F4/80^+^ macrophages from HFD mice (Figure 1C–D).

To investigate whether leptin and/or IL-6 mediate induction of ANXA1 in obese adipose tissue, we used leptin-deficient *ob/ob* mice and IL-6 KO mice on a C57BL6 background. Because *ob/ob* mice develop obesity even when fed chow diet, use of these animals also allowed us to investigate whether upregulation of ANXA1 requires feeding a HFD. Results shown in Fig. 1E and 1F indicate comparable upregulation of ANXA1 mRNA in VAT and SAT of both *ob/ob* mice on chow diet and IL-6 KO mice on HFD compared to their strain- and diet-matched WT controls. Thus, these data indicate that induction of ANXA1 in obesity is not dependent on HFD feeding and is not mediated by leptin or IL-6. Finally, we investigated whether upregulation of ANXA1 is observed in DIO-resistant female Balb/c mice, the strain we used for the functional studies described below. A significant two-fold increase in expression of ANXA1 mRNA was observed in VAT of female Balb/c mice fed HFD for 14 weeks compared to chow-fed controls. However, feeding a HFD did not lead to significant changes in expression of the ANXA1 receptor FPR2 in VAT of either C57BL6 or Balb/c mice (data not shown).

In summary, obesity in mice is associated with upregulated expression of ANXA1 in adipose tissue and this occurs in a leptin- and IL-6-independent fashion.

### Deficiency of ANXA1 modulates adiposity in mice

**Figure 2:**
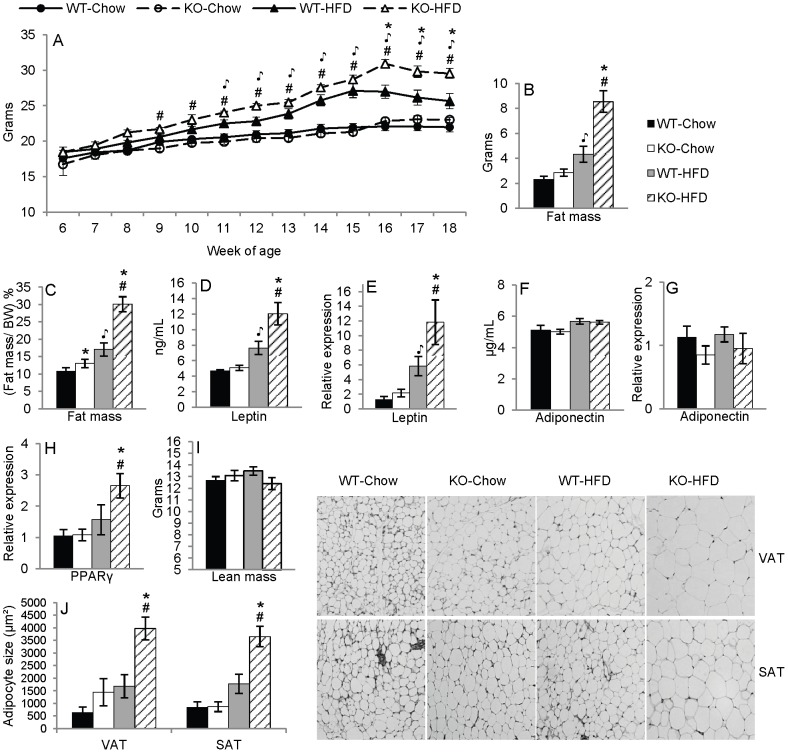
Deficiency of ANXA1 modulates adiposity in female Balb/c mice. Panel A: Body weight in grams, (n = 14 – 20). Panel B: Fat mass in grams measured by DXA. Panel C: Percentage of fat mass to BW (n = 14 – 19). Panel D: Plasma leptin levels (n = 13 – 17). Panel E: Leptin mRNA expression in VAT (n = 4 – 5). Panel F: Plasma adiponectin levels (n = 13 – 17). Panel G: Adiponectin mRNA expression in VAT (n = 5). Panel H: PPARγ mRNA expression in VAT (n = 4 – 5). Panel I: Lean mass in grams measured by DXA. Panel J: Median adipocyte size in VAT and SAT (n = 4 – 5), H&E-stained slides for VAT and SAT magnified to 10X. *p<.05 vs. respective diet-matched WT group, ^
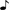
^p<.05 vs. WT-Chow, #p<.05 vs. KO-Chow. Data are mean ± SEM.

To test the hypothesis that ANXA1 deficiency will lead to increased adiposity due to the previously reported pro-lipolytic effects of ANXA1 [16], the obesity-resistant female Balb/c strain was used. Female Balb/c WT and ANXA1 KO mice were fed chow or HFD for 14 weeks. Body weight was measured weekly. As expected [19], feeding a HFD lead to only a modest increase in body weight in Balb/c WT mice, that became significant at 11 weeks of age (7 weeks of HFD), while weight became significantly increased in KO mice on HFD compared to those on chow at an earlier time point (9 weeks of age, 5 weeks of HFD) (Fig. 2A). By 16 weeks of age (12 weeks of diet) KO-HFD mice were significantly heavier than WT-HFD mice, whereas no differences were observed between chow-fed groups (Fig. 2A). No significant differences in food intake were observed between any of the groups (data not shown). Evaluation of body composition by DXA indicated a significant increase in fat mass in KO-HFD compared to WT-HFD mice, both in absolute terms and as a percentage of body weight (Fig. 2B–C), whereas no significant differences were observed between chow groups. The increased fat mass of KO-HFD mice was paralleled by elevated serum levels and VAT mRNA expression of leptin (Fig. 2D–E), confirming the presence of an expanded adipose tissue mass, with no differences among groups for serum or VAT mRNA expression of adiponectin (Fig. 2F), and a significant increase in mRNA expression of PPARγ (Fig. 2H). Additionally, body composition analysis by DXA revealed no differences in lean mass in grams between any of the groups (Fig. 2I), indicating that the increased body weight was selectively due to adipose tissue expansion rather than unspecific expansion of both lean and fat mass. Adipocyte size in VAT and SAT of KO-HFD mice was significantly higher compared to WT-HFD mice and to KO-chow mice, while no significant differences were found among the other groups (Fig. 2J), indicating development of adipocyte hyperplasia in ANXA1 KO mice fed HFD.

Overall, these data demonstrate that ANXA1 deficiency leads to increased adiposity in response to HFD in an obesity-resistant background.

### Effect of ANXA1 deficiency on lipolytic and lipogenic pathways

**Figure 3:**
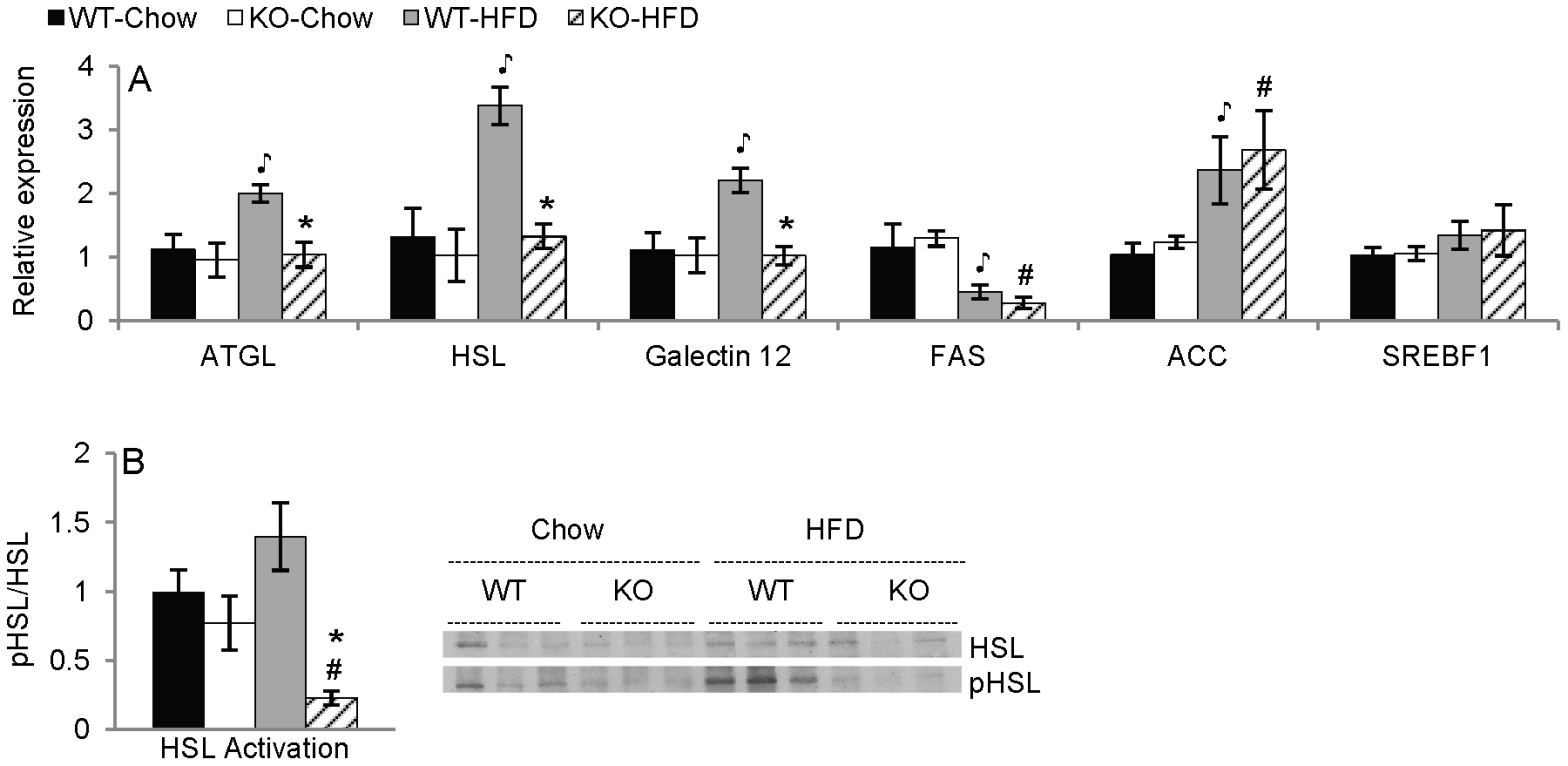
Expression of markers of lipolysis in VAT. Panel A: Expression of ATGL, HSL, and Galectin-12 mRNA in fasted mice and FAS, ACC, and SREBF1 mRNA in fed mice in VAT (n = 5). Panel B: Ratio of pHSL to HSL protein in fasted mice in VAT by western blot (n = 3). *p<.05 vs. respective diet-matched WT group, ^
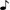
^p<.05 vs. WT-Chow, #p<.05 vs. KO-Chow. Data are mean ± SEM.

To evaluate mechanisms leading to increased adiposity in ANXA1 KO mice, we measured expression of genes associated with adipose tissue lipolysis, since this pathway was previously shown to be altered in the absence of ANXA1 [16]. A significant elevation in mRNA expression of the most important enzymes involved in adipose tissue lipolysis, i.e., ATGL, HSL, and Gal-12, was observed in VAT of fasted WT-HFD mice *versus* the respective chow groups (Fig. 3A). However, HFD did not lead to increased expression of these genes in KO mice, suggesting lack of compensatory upregulation of lipolytic pathways in the absence of ANXA1 (Fig. 3A). The difference between WT and KO mice was also evident at the protein level. In fact, the ratio of pHSL to total HSL protein was markedly reduced in fasted KO-HFD mice compared to each other group, indicating suppressed activation of this enzyme in KO mice in response to HFD (Fig. 3B).

We also wanted to investigate whether ANXA1 KO mice have altered lipogenic pathways. To this aim, we measured gene expression of two enzymes that control lipogenesis, FAS and ACC, and SREBF1, a transcription factor involved in lipid production. Feeding a HFD led to a significant downregulation of FAS and upregulation of ACC in VAT in both WT and ANXA1 KO mice (Fig. 3A), with no differences between strains. Moreover, no differences were observed for expression of SREBF1 among any of the groups. Thus, the lipogenic pathways we tested do not appear to be modified by ANXA1 deficiency.

These findings demonstrate that the increased adiposity of ANXA1 KO mice on HFD is associated with suppressed expression of genes involved in adipose tissue lipolysis, while ANXA1 deficiency does not appear to significantly affect lipogenic pathways in response to HFD.

### Altered glucose metabolism in ANXA1 KO mice

**Figure 4:**
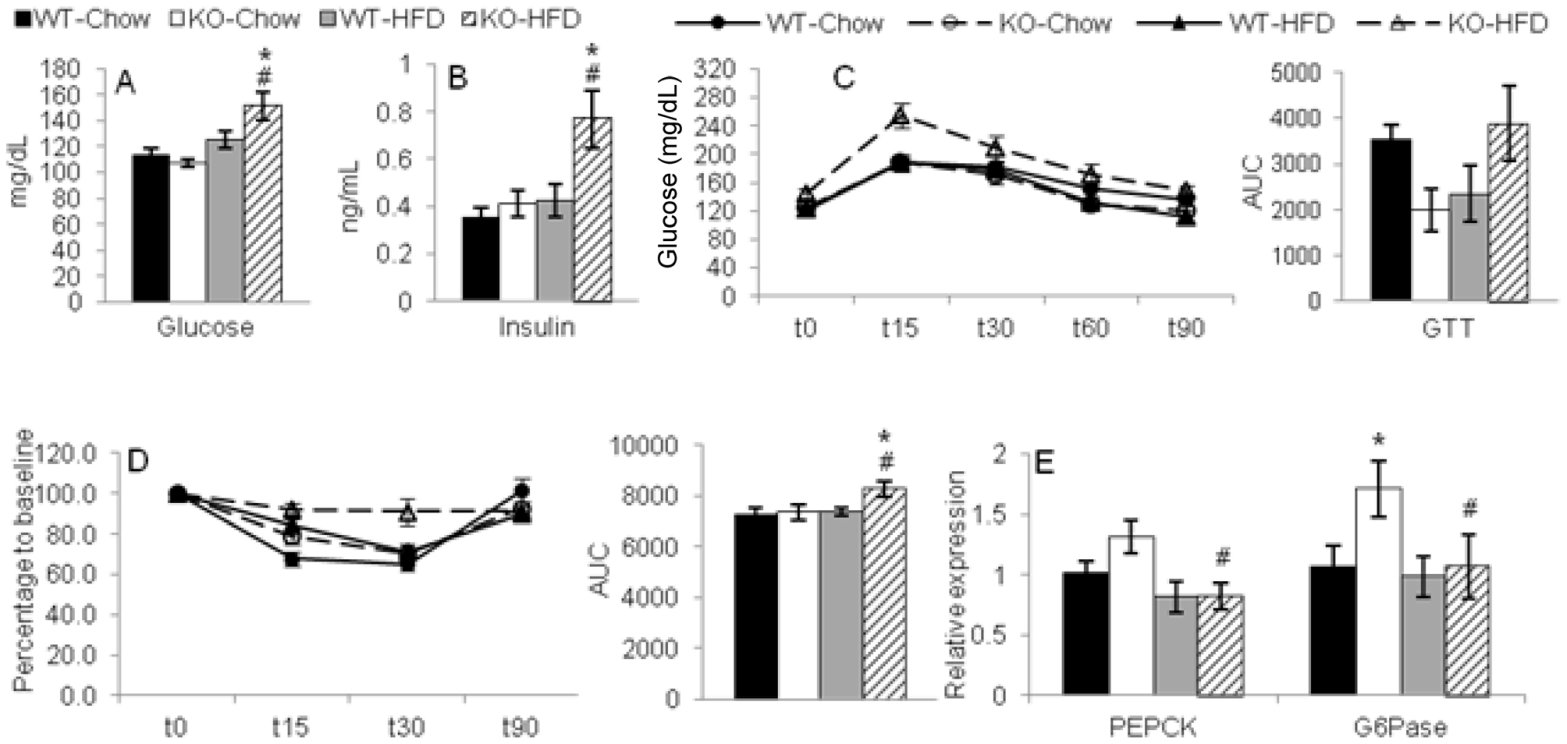
Dysregulated glucose metabolism in ANXA1 KO mice. Panel A: Fasting blood glucose (n = 14 – 20 per group). Panel B: Fasting plasma insulin (n = 9 – 15 per group). Panel C: Glucose tolerance test (GTT): change in blood glucose and area under the curve (AUC) (n = 3 – 5). Panel D: Insulin tolerance test (ITT): change in blood glucose as percentage to baseline and AUC (n = 3 – 5). Panel E: PEPCK and G6Pase mRNA expression in liver (n = 5). *p<.05 vs. respective diet-matched WT group, #p<.05 vs. KO-Chow. Data are mean ± SEM.

Because obesity is often associated with altered glucose metabolism, we next investigated whether ANXA1 deficiency affects glycemic control. Significantly higher fasting blood glucose and plasma insulin levels were observed in KO-HFD mice compared to each other group (Fig. 4A-B). Calculation of HOMA-IR, a measure of insulin resistance, indicated a significantly higher index in KO-HFD mice compared to the other groups (2.44±0.33, 2.71±0.35, 3.46±0.76 and 7.65±1.76 in WT-chow, KO-chow, WT-HFD and KO-HFD, respectively; p<0.01 in KO-HFD versus each other group; mean ± SEM; n = 4–6).

Evaluation of glucose tolerance by GTT, demonstrated a significant diet*strain effect by two-way ANOVA (p<0.05, Fig. 4C), with KO-HFD mice having the highest response, which indicates development of significant glucose intolerance in KO-HFD mice compared to the other groups. Moreover, KO-HFD mice were also significantly more insulin resistant compared to each other group as evaluated by ITT (Fig. 4D). However, no significant differences in hepatic expression of G6Pase and PEPCK, critical enzymes in the gluconeogenic response, were observed between WT-HFD and KO-HFD mice, although upregulation of these pathways was present in KO-Chow mice (Fig. 4E).

Overall, these data indicate the presence of deregulated glucose metabolism and insulin resistance in ANXA1 KO mice on HFD. However, because expression of gluconeogenic enzymes was not significantly altered in HFD ANXA1 KO mice compared to diet-matched WT mice, data suggest that the hyperglycemia of ANXA1 KO mice on HFD is unlikely to be the result of increased gluconeogenesis.

### Evaluation of GC metabolism in ANXA1 KO mice

**Figure 5:**
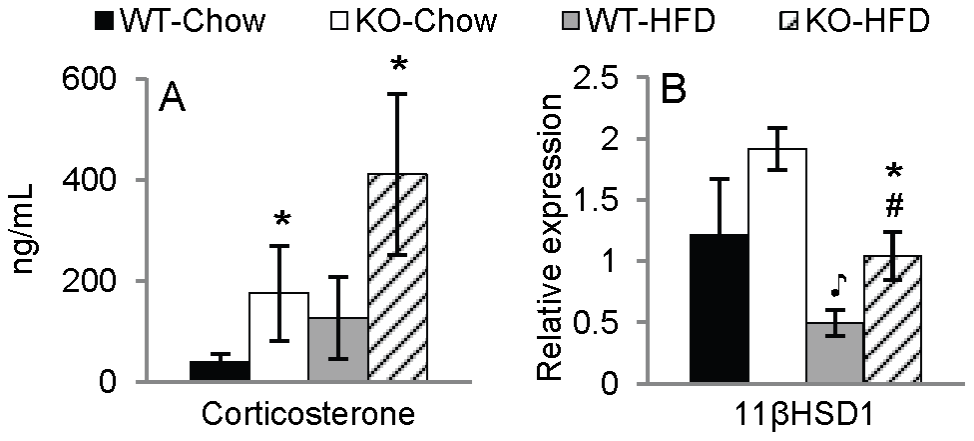
Corticosterone and 11βHSD1 levels in WT and ANXA1 KO mice. Panel A: Plasma corticosterone levels in fed mice (n = 5 – 13). Panel B: Expression of 11βHSD1 mRNA in VAT of fed mice (n = 4 – 5). *p<.05 vs. respective diet-matched WT group, ^
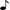
^p<.05 vs. WT-Chow, #p<.05 vs. KO-Chow. Data are mean ± SEM.

Chronic exposure to GC can lead to development of obesity [20]. Because ANXA1 is able to mediate the feedback inhibition of GC on release of adrenocorticotropic hormone [21]–[24], elevated levels of corticosterone as a result of lack of feedback inhibition of adrenocorticotropic hormone by GC may represent a potential mechanism leading to increased adiposity in ANXA1 KO mice. Indeed, ANXA1 KO mice on both chow and HFD had significantly elevated serum corticosterone levels compared to WT mice (Fig. 5A).

An additional GC-regulating pathway potentially involved in obesity is the enzyme 11βHSD1, which regulates intracellular levels of corticosterone [25]. In fact, increased expression of this enzyme in adipose tissue promotes adiposity [26], while obesity is associated with downregulation of its expression, probably as an attempt to control excess adiposity [27]. Feeding a HFD led to a significant reduction of 11βHSD1 expression in VAT of both WT and KO mice compared to strain-matched mice on chow (Fig. 5B). However, downregulation of this enzyme was blunted in VAT of KO-HFD, resulting in significantly higher levels of 11βHSD1 in KO-HFD compared to WT-HFD mice (Fig. 5B).

Thus, the presence of elevated corticosterone and blunted downregulation of 11βHSD1 represent additional potential mechanisms to explain the increased adiposity in ANXA1 KO mice.

### Evaluation of VAT inflammation in ANXA1 KO mice

**Figure 6:**
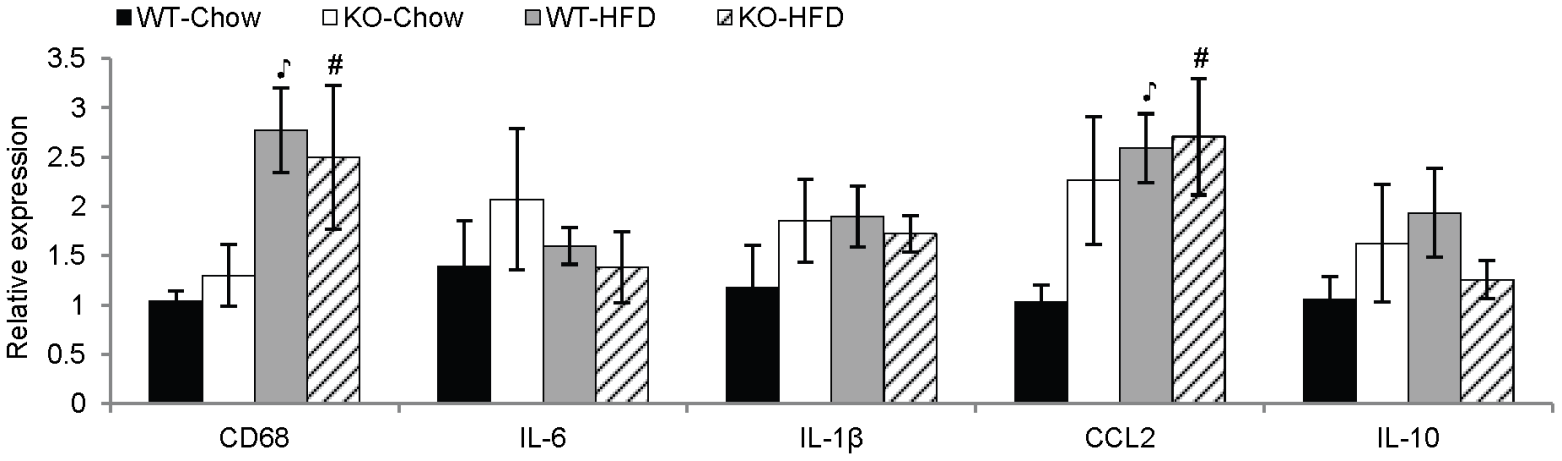
Markers of VAT inflammation in WT and ANXA1 KO mice. Expression of CD68, IL-6, IL-1β, CCL2, and IL-10 mRNA in VAT (n = 8 – 10). ^
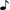
^p<.05 vs. WT-Chow, #p<.05 vs. KO-Chow. Data are mean ± SEM.

Because ANXA1 exerts several anti-inflammatory effects [28], we hypothesized that ANXA1 KO mice would have increased inflammation in VAT as compared to WT mice. As expected, feeding a HFD lead to significant elevation in gene expression of CD68, a marker of macrophage infiltration, and CCL2, a monocyte-attracting chemokine, in VAT compared to mice on chow (Fig. 6). However, no significant differences were observed when comparing KO mice to WT mice on either diet for expression of either CD68 or CCL2 and no significant diet*strain interactions were present. Expression of the pro-inflammatory cytokines IL-6 and IL-1β and the anti-inflammatory cytokine IL-10 in VAT was comparable among the four groups. Thus, no significant differences in VAT inflammation were observed between ANXA1 KO and WT mice.

## Discussion

In the present study we investigated regulation of ANXA1 expression in VAT of obese mice as well as the role of ANXA1 in modulating adiposity, pathways of glucose and fat metabolism and inflammation in response to HFD.

Data confirmed the main hypothesis of our study, revealing a significant increase in adiposity in HFD-fed ANXA1 KO mice compared to WT mice. The strength of these findings lies in the use of multiple measures of adiposity, including body composition by DXA, serum leptin levels and adipocyte size. However, it is important to note that WT and ANXA1 KO mice in the present study were not littermates, which could involve differences at epigenetic and microbiome levels. Our data are in conflict with results reported by Warne and colleagues [16], who demonstrated decreased epididymal fat pad mass in male C57BL6 ANXA1 KO mice on chow diet compared to WT mice. Thus, it will be important to perform future studies aiming at directly determining possible strain- and/or sex-differences in the role of ANXA1 in modulating adiposity.

In an attempt to elucidate the mechanisms by which ANXA1 modulates adiposity, we investigated multiple pathways, including enzymes involved in lipolysis and lipogenesis as well as hormonal regulation.

We observed upregulation of lipolytic pathways, including ATGL, HSL and Gal-12, in WT mice on HFD, in agreement with the published literature using the Balb/c strain [19], suggesting that Balb/c mice are able to compensate for increased energy intake by significantly upregulating lipolysis. This mechanism is lacking in the obesity-prone C57BL6 strain, in which HFD instead leads to suppressed levels of these proteins [19], [29]. Interestingly, our data indicate the presence of an intermediate phenotype in Balb/c ANXA1 KO mice, in which HFD failed to modulate ATGL, HSL or Gal-12 in either direction, thus supporting a role for ANXA1 in regulating lipolysis-associated pathways in VAT.

In contrast, ANXA1 did not appear to be involved in modulation of lipogenic pathways in response to HFD, as demonstrated by lack of differences between WT and KO mice for expression of FAS, ACC and SREBF1. However, a more detailed functional analysis of both lipolytic and lipogenic pathways will be necessary to conclusively demonstrate the involvement on ANXA1 in lipid metabolism and the mechanism mediating the observed effects.

We also found significantly elevated plasma corticosterone levels in ANXA1 KO mice compared to WT mice on both chow and HFD. Because chronic exposure to or upregulation of GC leads to increased adiposity [30], these results suggest that ANXA1 deficiency may promote adiposity through its known role in modulating the hypothalamus-pituitary-adrenal axis. Moreover, we demonstrated blunted downregulation of 11βHSD1 in response to HFD in ANXA1 KO mice compared to WT mice, suggesting a role for ANXA1 in modulating levels of this enzyme. Since over-expression of 11βHSD1 leads to increased adiposity, elevated leptin levels, insulin resistance and hyperlipidemia [26], while a selective 11βHSD1 inhibitor prevents adipogenesis [31], alteration of this pathway by ANXA1 represents an additional potential mechanism to explain the increased adiposity of KO-HFD mice.

At variance with WT mice, ANXA1 KO mice on HFD developed significant, although mild, fasting hyperglycemia and hyperinsulinemia – coupled with insulin resistance and glucose intolerance. Since insulin resistance only occurred in ANXA1 KO mice on HFD but not on chow, it is likely this phenotype was secondary to increased adiposity rather than being independently caused by ANXA1 deficiency. Assessing adiposity and insulin resistance throughout the life-time in future studies will help in confirming the time sequence in which these metabolic events take place. Although hyperinsulinemia was previously reported in male C57BL6 ANXA1 KO mice on chow diet [16], in our study chow-fed female Balb/c ANXA1 KO mice only showed a non-significant trend towards elevated plasma insulin, a discrepancy likely attributable to strain- and sex-specific differences.

Because ANXA1 is an anti-inflammatory protein, one would expect that deficiency of this gene would lead to development of increased adipose tissue inflammation in response to HFD. Surprisingly, no differences in markers of VAT inflammation were observed when comparing ANXA1 KO mice to WT mice. This is even more remarkable when considering that ANXA1 KO mice developed increased adiposity and insulin resistance in response to HFD, which should have been associated with heightened inflammation. However, it should be noted that mice of the Balb/c strain develop only minor VAT inflammation in response to HFD. Therefore, evaluating the effect of ANXA1 deficiency in VAT inflammation in an obesity-prone strain may generate more conclusive results of the functional association between ANXA1 and metabolic inflammation. In agreement, we show that HFD-induced upregulation of ANXA1 expression was much more pronounced in adipose tissue of obesity-prone C57BL6 mice compared to Balb/c mice. Elevated expression of ANXA1 in adipose tissue of obese mice parallels the increased ANXA1 expression observed in adipose tissue of obese humans [12], but contrasts with the attenuated plasma levels of ANXA1 reported in human obesity [13]. It is possible that ANXA1 is sequestered inside the expanding adipose tissue in obesity, preventing its release into the circulation. In support of this notion, we observed significantly increased cell-associated ANXA1protein in VAT macrophages of HFD mice, indicating that increased mRNA expression is paralleled by elevated protein production. In future studies it will be useful to evaluate adipose tissue expression, macrophage-associated protein as well as circulating levels of ANXA1 in parallel.

## Conclusion

While female Balb/c mice are resistant to DIO and the associated insulin resistance, we demonstrate that deficiency of a single protein, ANXA1, significantly alters the metabolic profile of this strain towards augmented susceptibility to weight gain. Thus, we present compelling evidence that ANXA1 is an important regulator of adiposity in mice, possibly through regulation of lipolysis and/or GC levels.
